# Experiences of fear in hospital settings from the perspectives of mental health service users and informal caregivers

**DOI:** 10.1192/j.eurpsy.2021.355

**Published:** 2021-08-13

**Authors:** T. Lantta, M. Anttila, J. Varpula, M. Välimäki

**Affiliations:** 1 Department Of Nursing Science, University of Turku, UNIVERSITY OF TURKU, Finland; 2 Xiangya School Of Nursing, Central South University, Changsha, China

**Keywords:** Psychiatric hospital, Fear, Service user, Informal caregiver

## Abstract

**Introduction:**

In the literature, service users and informal caregivers have been critical towards psychiatric inpatient care. However, little is known about their fears related to hospital care.

**Objectives:**

We describe service users’ and informal caregivers’ experiences of fear in psychiatric hospital settings.

**Methods:**

The data were collected from seven mental health associations located in six Finnish cities. Focus group interviews (f=8) were conducted (2015–2016) with service users (n=20) and informal caregivers (n=15), and were guided to focus on violence and challenging situations in psychiatric care. In a secondary analysis, experiences of fear were extracted from the transcriptions and analyzed using inductive content analysis.

**Results:**

Both groups’ experiences of fear focused on themes related to staff, treatment and fellow patients. Additionally, service users had experiences of fear related to the hospital environment. Fears related to staff involved intimidating personnel using force or acting in threatening ways. Participants also described staff seemingly being afraid of patients and care givers. Three types of fears related to treatment were described: fear of not being admitted to hospital even if needed, fear of being admitted to hospital, and fear of coercive methods used in care. Fear of fellow patients involved being afraid of aggressive, unpredictable behaviors, which could cause, e.g., a lack of sleep at night for service users. Fears related to the environment itself were also discussed.

**Conclusions:**

Being hospitalized can be a difficult experience for service users and informal caregivers. These results can help psychiatric healthcare staff acknowledge areas in care that may potentially cause feelings of fear.

**Disclosure:**

No significant relationships.

